# Field swimming performance of bluegill sunfish, *Lepomis macrochirus*: implications for field activity cost estimates and laboratory measures of swimming performance

**DOI:** 10.1002/ece3.3454

**Published:** 2017-09-30

**Authors:** Kelsey Cathcart, Seo Yim Shin, Joanna Milton, David Ellerby

**Affiliations:** ^1^ Department of Biological Sciences Wellesley College Wellesley MA USA

**Keywords:** field behavior, kinematics, *Lepomis macrochirus*, metabolic rate, swimming

## Abstract

Mobility is essential to the fitness of many animals, and the costs of locomotion can dominate daily energy budgets. Locomotor costs are determined by the physiological demands of sustaining mechanical performance, yet performance is poorly understood for most animals in the field, particularly aquatic organisms. We have used 3‐D underwater videography to quantify the swimming trajectories and propulsive modes of bluegills sunfish (*Lepomis macrochirus*, Rafinesque) in the field with high spatial (1–3 mm per pixel) and temporal (60 Hz frame rate) resolution. Although field swimming trajectories were variable and nonlinear in comparison to quasi steady‐state swimming in recirculating flumes, they were much less unsteady than the volitional swimming behaviors that underlie existing predictive models of field swimming cost. Performance analyses suggested that speed and path curvature data could be used to derive reasonable estimates of locomotor cost that fit within measured capacities for sustainable activity. The distinct differences between field swimming behavior and performance measures obtained under steady‐state laboratory conditions suggest that field observations are essential for informing approaches to quantifying locomotor performance in the laboratory.

## 1 INTRODUCTION

Locomotion is vital to the survival and fitness of most animals. Mobility is required for effective foraging, predator avoidance, migration, and for many social interactions (Miles, 2004; Walker, Ghalambor, Griset, McKenney, & Reznick, 2005; Watkins, 1996). The costs of locomotion can also dominate daily energy budgets (Boisclair & Sirois, 1993; Irschick & Garland, 2001; Kerr, 1982). These costs are primarily associated with the muscle activity needed to generate propulsive forces or maintain stability (Gerry & Ellerby, 2014; Marsh & Ellerby, 2006). The majority of the available data concerning mechanical performance and energy cost during locomotion were obtained under quasi steady‐state conditions with linear motion and repeated, regular propulsive cycles (Brett, 1964; Hoyt & Taylor, 1981; Tucker, 1966). In contrast, the volitional locomotor behavior of most animals may be intrinsically unsteady, with nonlinear motion and irregular propulsive movements (Biewener & Daley, 2007; Fuiman & Webb, 1988; Kramer & McLaughlin, 2001). This mechanical variability requires greater muscle force and power output, and therefore more metabolic energy than under steady‐state conditions at the same speed (Daniel, 1984; Webb, 1991). This calls into question the utility of steady‐state performance data for estimating the activity component of field metabolic rate (FMR) or establishing the links between particular performance traits and organismal fitness.

The connection between laboratory performance data and the field can only be validated with reference to field motion data with a high spatial and temporal resolution (Broell et al., 2013). Temporal resolutions of <10 Hz may not detect changes in locomotor behavior (Broell et al. (2013), and minimum spatial resolutions in the order of 10% of body length or wing span are needed to estimate center of mass location and trajectory (Theriault et al., 2014). This level of detail may not be available from telemetry of physiological or mechanical parameters such as heart rate or body acceleration that serve as proxies for activity level or behavior (Lucas, Johnstone, & Priede, 1993; Standen, Hinch, Healey, & Farrell, 2003; Ward, Bishop, Woakes, & Butler, 2002; Webber, Boutilier, Kerr, & Smale, 2001; Webber & O'Dor, 1986) or motion tracking techniques such as acoustic telemetry, sonar, or GPS (Cooke et al., 2005; Hanson, Hasler, Donaldson, & Cooke, 2010; de Kerckhove, Milne, & Shuter, 2015; Wilson et al., 2013). Video analyses allow reconstruction of animal trajectories in three dimensions (Krohn & Boisclair, 1994; Theriault et al., 2014) and have the added advantage of directly revealing the mode of propulsion, rather than an associated mechanical signature. This final component is important, as a focus purely on performance metrics such as average velocity could mask functionally significant differences in the underlying behavior that affect metabolic cost.

In order to assess the applicability of laboratory performance data to volitional field behavior and the estimation of field metabolic rate (FMR), we have undertaken a video analysis of field swimming behavior in bluegills sunfish (*Lepomis macrochirus*, Rafinesque). Although previous studies have used stereo‐videography to characterize volitional swimming behavior in fish (Boisclair & Tang, 1993), this is the first field video study with sufficient temporal and spatial resolution (Broell et al., 2013) to fully characterize the mechanical details of field swimming in a fish species. Bluegills sunfish are ideal for a laboratory–field comparison as there is a large body of laboratory data concerning their swimming performance, energetics, and swimming muscle properties that allow a clear comparison to be made between field and laboratory locomotor behavior (Drucker & Lauder, 1999; Jayne & Lauder, 1993; Jones, Jong, & Ellerby, 2008; Kendall, Jones, Lucey, & Ellerby, 2007). This will indicate whether steady‐state cost–speed relationships can be applied to estimate the activity component of FMR based on field swimming velocities, or whether the basic mechanical characteristics of field swimming differ from those observed during steady‐state flume swimming, precluding application of the associated cost data to the field. If the latter case applies, our data will allow a further assessment of whether existing models that incorporate a “volitional cost” increment to account for unsteady swimming provide reasonable estimates of field activity costs in this and other species, or whether an alternate approach can be adopted. Steady‐state locomotor cost data are available for a wide range of fish species, more so than in any other vertebrate group (Videler, 1993). If an approach to estimating the cost increment associated with the unsteadiness characteristic of field locomotion can be applied, this raises the possibility of enhancing FMR estimates in a diversity of fish species.

## 2 MATERIALS AND METHODS

**Figure 1:**
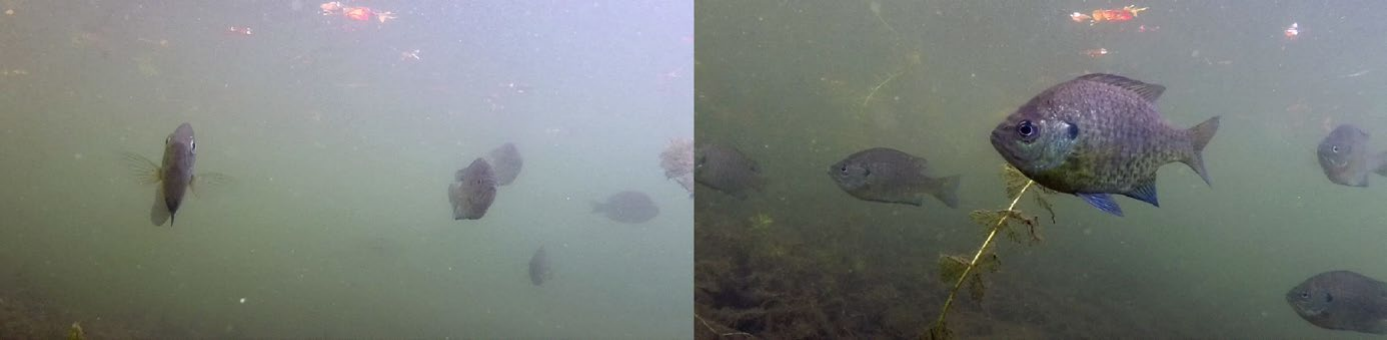
Simultaneous stereo video stills of bluegill sunfish (*Lepomis macrochirus*) in Lake Waban, MA, USA. Images were captured using a pair of GoPro Hero3 Silver cameras. The volume imaged by both cameras was calibrated using a direct linear transform method to allow 3‐D reconstruction of fish trajectories

**Figure 2:**
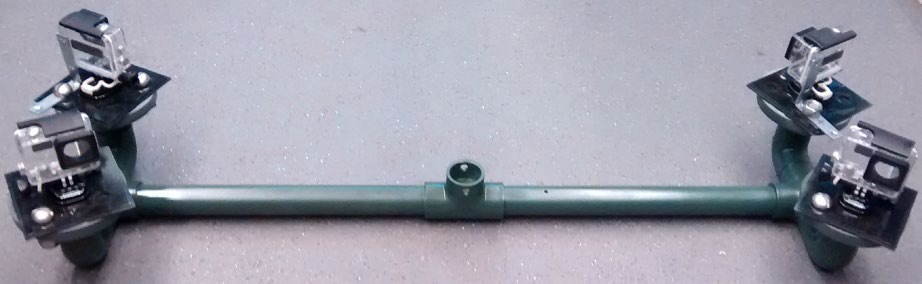
Camera head consisting of four GoPro underwater video cameras, mounted in pairs with overlapping fields of view to allow three‐dimensional reconstructions of fish swimming trajectories

Video was collected in Lake Waban, Massachusetts, USA, in August and September 2015 at a 60 Hz frame rate with four GoPro video cameras (GoPro, Hero 3 Silver, San Mateo, CA, USA). These were mounted in pairs on a camera head with overlapping fields of view (Figures 1 and 2). Cameras were deployed in locations with approximately 1 m water depth, and the camera head located 50 cm below the water surface. Water temperature at the camera location was measured with a thermocouple temperature probe. Analyzed video sequences were collected between 11 a.m. and 2 p.m. EST. The first 30 min of video after camera deployment were discarded to minimize disturbance effects from camera placement. Performance data were obtained from 46 swimming tracks. The mean track duration was 21.6 s, and mean track length was 1.2 m. Mean total fish length was 0.18 ± 0.04 m. Fish lengths were estimated from the video images by tracking the snout and tail trailing edge positions on a minimum of 10 frames for a given individual and taking the average of the snout–tail distances as the length for that fish. For bluegills sunfish, body mass, *M*, kg = 19.95*L* ^3^, where *L* is total body length in m. On this basis, mean estimated mass was 0.116 ± 0.001 kg. The 46 tracks may represent data from <46 individuals. However, given the temporal and spatial distribution of sampling across nine deployment days and three littoral locations, and a likely population of several thousand individuals in the size range observed (based on lake area and population density estimates from comparable lakes, Osenberg, Werner, Mittelbach, & Hall, 1988; Schneider, 2000), the probability of resampling was low.

Camera pairs were calibrated using a wand calibration technique and the direct linear transform (DLT) method (Tsai, 1987). Videos of wand movements defined a series of coordinates throughout the imaged volume based on wand end positions and were used to generate 11 DLT coefficients for each camera pair (Theriault et al., 2014). Lens distortion coefficients were also quantified using a checkerboard imaging technique (Bouguet, Camera Calibration Toolbox for MATLAB, http://www.vision.caltech.edu/bouguetj/index.html). Fish center of mass (COM) locations were tracked through the calibrated volumes with a MATLAB‐based digitizing program (MATLAB 2014a, The Mathworks Inc., Natick, MA, USA) developed by Hedrick (2008). The COM of bluegills is located approximately 40% of total body length from the snout (Gerry, Robbins, & Ellerby, 2012). This landmark position was estimated visually from the video images. Position data were smoothed using a smoothing spline interpolation in the application Igor Pro (v. 6.2, Wavemetrics, Lake Oswego, OR, USA). This method is similar to the cubic spline algorithm recommended by Walker (1998) for calculating velocities and accelerations from position data. The level of smoothing applied by the algorithm was defined by the standard deviation of the data. Smoothed COM position–time data were differentiated to obtain COM velocity, and velocity was differentiated to obtain COM acceleration. Smoothing filters out small stochastic errors in estimating the center of mass location that arise from image pixelation. In the absence of smoothing, these errors are magnified during the first‐ and second‐order differentiations to obtain velocity and acceleration estimates (Walker, 1998). Perpendicular velocity vector magnitudes were used to calculate the tangents of heading angles. The tangent of the path angle in the horizontal plane was calculated as the ratio of the perpendicular velocity vector magnitudes in the horizontal plane. The tangent of the vertical path angle was calculated as the ratio of the resultant horizontal and the vertical velocity vector magnitudes. Path angles were calculated as the arctangents of the velocity vector ratios. Angles were differentiated with respect to time to determine horizontal (yaw) and vertical (pitch) angular velocities. Path radius of curvature relative to body length (*L*) was calculated as the center of mass velocity in body lengths per second (*L/*s) divided by the angular velocity (radians/s).

Fish behavior during each video track was categorized. Bluegills exhibit two swimming gaits: median paired fin (MPF) swimming powered by pectoral fin movements; and body caudal fin (BCF) swimming powered by undulations of the body axis and caudal fin (Kendall et al., 2007). BCF swimming may be sustained or intermittent, where a single “kick” tailbeat is followed by a period of nonpropulsive gliding. Behavior was placed into the following categories: station holding with no detectable COM motion, MPF swimming; BCF swimming consisting of three or more consecutive tail beats; single “kick” tailbeats; and nonpropulsive gliding.

A baseline field metabolic rate was estimated using the relationship between bluegills aerobic metabolic rate and swimming speed previously established during flume swimming for fish in this size class at the approximate water temperature measured during field recordings (22.4 ± 0.5°C, mean ± *SD*). In bluegills, aerobic metabolic rate during flume swimming, *M*_flume_, W/kg = 0.46 + 0.23*U* ^2.75^ (Kendall et al., 2007), where *U* is speed in body lengths per second (*L/*s). The cost relationship will only reflect this total mechanical power requirement if there is no anaerobic contribution to metabolic power. The maximal aerobically supported swimming speed of bluegills sunfish is 2.14 *L/*s (Kendall et al., 2007; D. Ellerby unpublished data, average fish mass 0.104 kg), above which some metabolic power must be supplied anaerobically. This is indicated by excess postexercise oxygen consumption (EPOC), elevated energy expenditure associated with a range of physiological processes including lactate clearance and glycogen synthesis (Milligan, 1996; Peake & Farrell, 2004; Svendsen, Tirsgaard, Cordero, & Steffensen, 2015; Svendsen et al., 2010). The cost–speed relationship being applied was obtained across a speed range designed to exclude anaerobic effort (Kendall et al., 2007; Ellerby & Gerry 2011). However, if EPOC costs are included with aerobic energy expenditure during swimming, the total cost continues to follow the curvilinear cost–speed relationship obtained for purely aerobic activity (Lee et al., 2003; Svendsen et al., 2015). Swimming costs for velocities above the aerobic speed limit for bluegills were therefore estimated by extrapolation of the aerobic cost–speed relationship to the measured velocity. Bouts of active propulsion were commonly interspersed with gliding. If this was the case, the cost–speed relationship was only applied to periods of active propulsion. The relationship spans MPF and BCF gaits and was applied to both forms of propulsion. For nonpropulsive gliding phases and for station holding, the metabolic rate was assumed to equal the zero‐speed intercept value of the cost–speed relationship (0.46 W/kg, Kendall et al., 2007).

**Figure 3:**
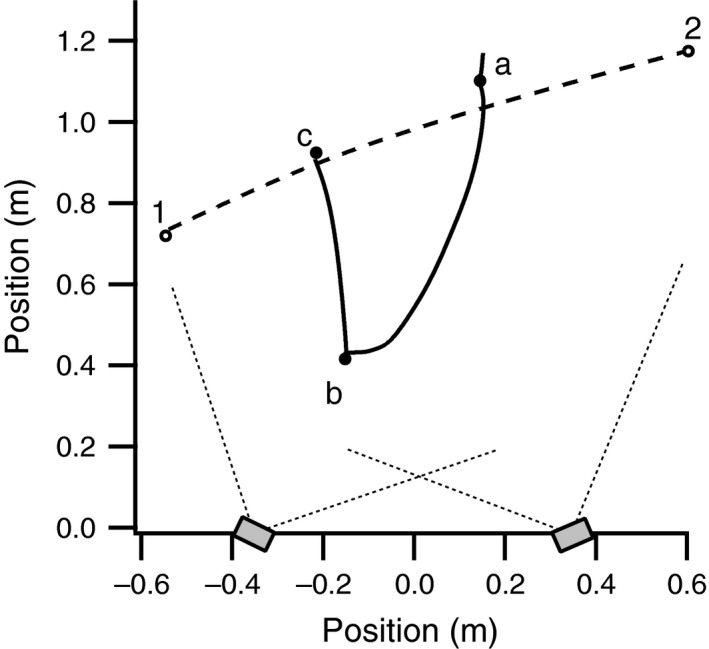
Example fish trajectories. Trajectories are shown in two dimensions within the horizontal plane with respect to a camera pair. Approximate camera positions and horizontal fields of view are indicated by gray boxes and dotted lines. The dashed line indicates a simple trajectory where a fish followed a curved path through the imaged volume. The unbroken line indicates a more complex track where a fish engaged in bouts of station holding at locations a, b, and c

Maneuvers that require changes in trajectory or momentum must elevate the costs of swimming above those determined under steady‐state conditions (Webb, 1991). If field acceleration and deceleration rates are generally low, then turning will be the primary mechanical factor that elevates field metabolic rate above that obtained under steady‐state flume conditions. For a neutrally buoyant fish doing additional mechanical work to maintain a curved swimming path, the relative elevation in cost above that of straight line swimming can be calculated as follows (Weihs, 1981):

(1) $$\frac{M_{\mathrm{curve}}}{M_{\mathrm{flume}}}\;=\;\sqrt{1\;+\;2\frac{V^{1/3}}{{\text{RC}}_{D}}{\left(\frac{\rho f}{\rho w}\;+\;\lambda \right)}^{2}}$$

where *M*_curve_ is the metabolic cost of swimming a curved path, *M*_flume_ is the measured metabolic cost of linear flume swimming, *V* is the estimated volume of the fish in m^3^, *R* is the radius of path curvature in m, *C*_*D*_ is the total drag coefficient of the fish, ρ_*f*_ and ρ_*w*_ are the densities of fish and water, respectively, in kg m^3^, and λ is the added mass coefficient. *C*_*D*_ is a dimensionless coefficient that summarizes the drag force inducing properties of a body in motion through a fluid and is related to drag force as follows:

(2) $$C_{D}\;=\;\frac{2D}{\rho U^{2}A}$$

where *D* is the drag force in N, ρ is fluid density in kg/m, *U* is velocity in m/s, and *A* is a reference area (typically total surface area) in m^2^. Computational methods place the *C*_*D*_ in the 0.05–0.1 range for swimming fish of a comparable size and velocity (Ahlborn, Harper, Blake, Ahlborn, & Cam, 1991; Schulz & Webb, 2002). The added mass coefficient, λ*,* is a further dimensionless coefficient. It accounts for the fact that a body in motion through a fluid also induces motion in that fluid, in effect increasing the mass in motion beyond that of the body mass of the fish alone. For a fish‐shaped body, λ is approximately 0.2 (Webb, 1975). Given the uncertainty regarding the magnitude of *C*_*D*_, cost increments were calculated using a *C*_*D*_ of both 0.05 and 0.1 to define the likely range of costs. Example swimming tracks are shown in Figure 3. For simple, curved tracks, the metabolic rate, estimated from the associated flume swimming cost at the corresponding velocity, was multiplied by the cost increment associated with the average radius of curvature of the track. For more complex tracks, for example, those interrupted by bouts of station holding (Figure 3), cost was estimated for segmentally using the associated velocities and average radii for each subsection of the track.

## 3 RESULTS

**Figure 4:**
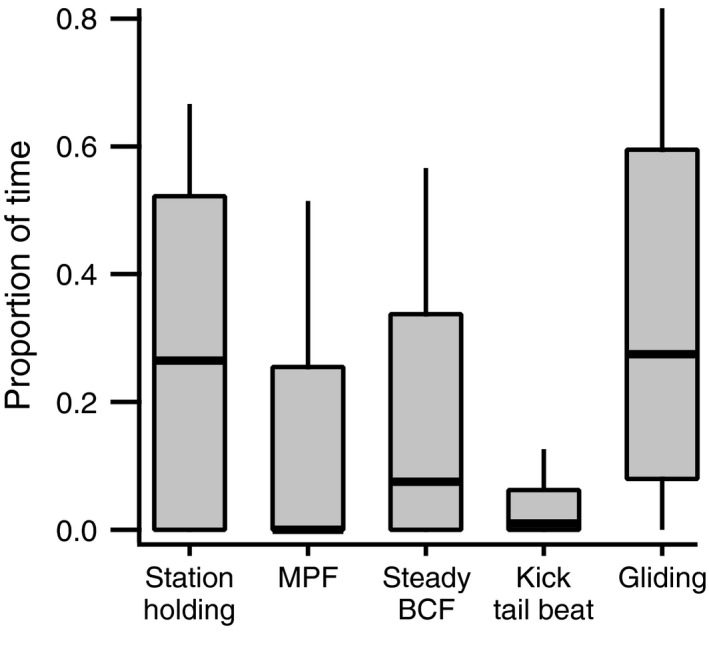
Proportion of total time spent engaged in specified behaviors by bluegills sunfish during routine field swimming. Data were taken from 46 tracked swimming sequences. Bold, horizontal lines show median values, boxes the 25th and 75th percentiles, and whiskers the 10th and 90th percentiles

Fish generally held station in, or moved slowly through the imaged volumes. Transient accelerations to capture food items were also observed, but in only 4 of 46 tracked swimming sequences. The proportion of time spent on particular swimming behaviors during routine swimming is shown in Figure 4. The median value for time spent on station holding was 26.5%. A large proportion of the total observed time was also spent on variants of BCF swimming. This was dominated by the inactive glide component of intermittent kick and glide swimming, with a median of 27.5% of the total time (Figure 4).

**Figure 5:**
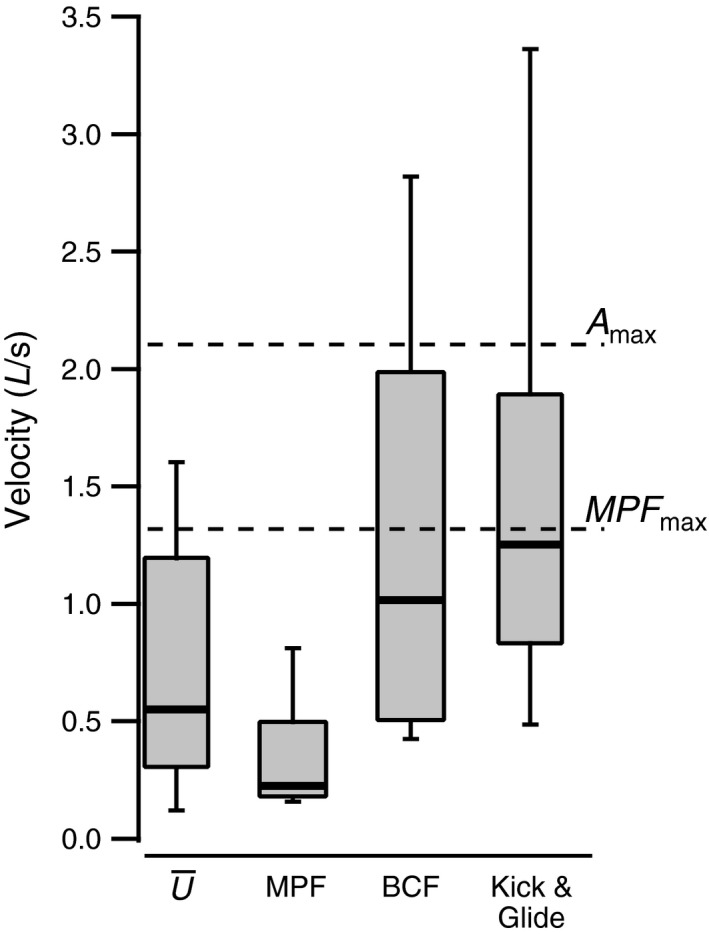
Bluegills sunfish field swimming velocities. The overall average velocity $\overset{¯}{U}$ includes periods of station holding. Bold, horizontal lines show median values, boxes the 25th and 75th percentiles, and whiskers the 10th and 90th percentiles. Data were taken from 46 tracked swimming sequences. For kick and glide swimming, the velocity is averaged across both the kick and glide phases. *N* = 46, 57, and 61 for MPF, BCF, and kick and glide observations, respectively. Differences in sample sizes between number of tracks and number of observed behaviors reflect the potential for several bouts of a particular behavior to arise within a single tracked sequence. Maximal velocities for MPF and aerobic BCF flume swimming are shown for comparison (data from Kendall et al., 2007)

Speed averages taken across the duration of each tracked sequence were skewed heavily to low values (Figure 5). This was due to the high proportion of total time spent on station holding or low‐speed MPF swimming by some individuals (Figure 4). MPF swimming was employed at low speeds, but not across the potential range of swimming velocities it can support during flume swimming (Figure 5). BCF and kick and glide swimming were both used to support a wide range of swimming velocities (Figure 5). These were generally within the speed range supported by aerobic metabolism during flume swimming (Figure 5).

**Figure 6:**
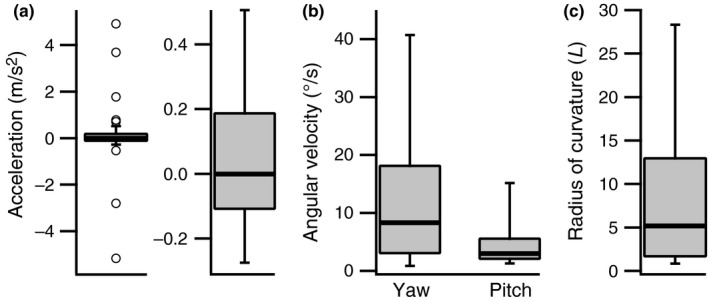
Unsteady and nonlinear aspects of field swimming behavior in bluegills sunfish. (a) Acceleration. (b) Average angular velocities in yaw and pitch. (c) Average path radius of curvature. Bold, horizontal lines show median values, boxes the 25th and 75th percentiles, and whiskers the 10th and 90th percentiles. Acceleration data are shown both with and without outliers to enable both the full range of values to be clearly visualized. Data were taken from 46 tracked swimming sequences

Accelerations and decelerations were modest, well within the maximum scope for this species which approaches 200 *L*/s^2^ during escape responses or maneuvers around obstacles (Ellerby & Gerry, 2011; Webb, 1978), and also within the range measured during sustained flume swimming at an applied average velocity in other species (*Galaxias maculatus*, Plew, Nikora, Larned, Sykes, & Cooper, 2007; *Acipenser fulvescens*, Thiem et al., 2015). A wide range of average path radius of curvature values was calculated, with a median value of 5.2 *L* (Figure 6).

**Figure 7:**
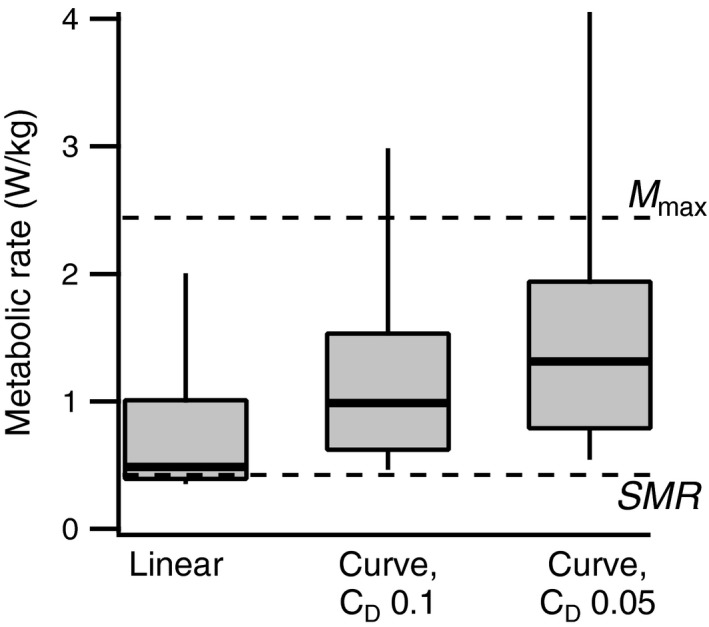
Estimated bluegills sunfish field metabolic rates. Metabolic rates predicted from measured velocities based on flume swimming cost measurements. Metabolic rates predicted from a factorial cost increment to linear swimming costs based on path curvature and assuming a drag coefficient of 0.05 or 0.1. The maximum aerobic metabolic rate during flume swimming, *M*_max_, and the zero‐speed intercept of the cost–speed relationship are indicated by the horizontal dashed lines (data from Kendall et al., 2007). Station holding and the gliding component of swimming were assigned a cost equivalent to this zero‐speed intercept

Estimates of FMR derived from flume swimming energy expenditure data and measured field velocities and path curvatures (Figures 5 and 6) are shown in Figure 7. Median estimated FMR based on flume swimming energetics at an equivalent velocity was 0.49 W/kg, equivalent to 20% of the aerobic maximum. Median FMR estimates incorporating path curvature ranged from 0.99 to 1.31 W/kg or 41% to 55% of the aerobic maximum.

## 4 DISCUSSION

### 4.1 Applying laboratory data to the field

Laboratory estimates of swimming cost can only be applied to estimate the activity component of FMR if laboratory and field locomotor behaviors are mechanically similar. Flume swimming is linear and typically carried out at a constant average velocity (Brett, 1964; Lee et al., 2003; Ellerby & Herskin, 2013; for an exception see Roche et al., 2014) and is therefore a poor match for the swimming behaviors observed in the field (Figure 6). Existing energetic data for volitional swimming were also obtained under conditions that did not resemble field behavior in bluegills. For example, average angular velocities of 19–64/°s in volitional swimming cost studies (Tang & Boisclair, 1995; Krohn & Boisclair, 1994; Tang, Boisclair, Menard, & Downing, 2000; Steinhausen, Steffensen, & Andersen, 2010) exceeded averages in the present study (14.7/°s in yaw, and 5.8/°s in pitch, Figure 6). The actual disparity is likely greater than this as previous energetic studies divided fish trajectory data into 1 s segments, equivalent to a 1 Hz sampling rate for performance estimates, compared with 60 Hz in the present study. Down sampling our position data to 1 Hz gave angular velocity estimates of <3/°s in yaw and 2/°s in pitch. This may be a consequence of intrinsic interspecific differences in swimming behavior as the comparable volitional energetic data were obtained for gilthead seabream (*Sparus aurata*, Steinhausen et al., 2010) and brook trout (*Salvelinus fontinalis*, Tang & Boisclair, 1995; Krohn & Boisclair, 1994; Tang et al., 2000), but small turning radii imposed by the enclosures used are likely an important factor. The available energetic data are therefore snapshots from opposite ends of a spectrum of mechanical unsteadiness and linearity, neither of which may directly inform estimates of FMR. The mechanical mismatch is important as it means that neither flume swimming data or existing models for field activity cost based on volitional energetic data (Boisclair & Sirois, 1993; Boisclair & Tang, 1993; Tang et al., 2000) are applicable to our field data. A further, more general concern is that volitional cost models produce cost estimates that may exceed the metabolic capacities of many fish species. For example, estimated factorial cost elevations (ratio of volitional to steady‐state energy costs) for field relative to steady‐state costs range from three‐ to 22‐fold (Boisclair & Tang, 1993; Tang et al., 2000) and indicate a factorial of 6.6 for the observed median field speed and mean body mass for bluegills (Boisclair & Tang, 1993). This would place bluegills beyond their limit for sustained aerobic effort indicated by their factorial aerobic scope (FAS), the ratio of maximal aerobic to standard metabolic rate (Kendall et al., 2007), requiring the use of anaerobic energy sources. This overestimation may apply in other teleost fish where FAS typically ranges from 2 to 10 (Clark, Sandblom, & Jutfelt, 2013; Killen, Costa, Brown, & Gamperl, 2007).

In the absence of energetic data that more closely replicate field swimming mechanics, an alternate approach based on swimming trajectory could be adopted. Given that acceleration rates during volitional swimming were modest (Figure 6), and straight swimming trajectories were rare, the added energetic costs of turning are the major factor likely to elevate cost above steady‐state levels. The relative cost of turning in fish is a function of path curvature and is largely independent of velocity (Weihs, 1981). The factorial increase is in part dependent on the drag coefficient of the fish. This is not well quantified for actively swimming fish, so values that span the range of available estimates were used. The median costs predicted from path curvatures were 2.0‐ to 2.7‐fold higher than those based purely on swimming velocity for a drag coefficient range of 0.05–0.1. These yielded swimming cost estimates that were generally within the measured aerobic scope for bluegills sunfish (Figure 7). This approach may therefore provide more attainable cost estimates for the observed swimming behaviors than existing energetic models.

### 4.2 Implications for laboratory studies of swimming performance

If laboratory performance data are to be linked to organismal function and fitness in the field, then experimental conditions must produce behaviors that are a reasonable analog for those observed in the field (Hertz, Huey, & Garland, 1988; Irschick, 2003; Irschick, Herrel, VanHooydonck, Huyghe, & Van Damme, 2005; Nathan et al., 2008; Vanhooydonk & Van Damme, 2003). The logical first step in this process is quantifying field performance. Despite this, there is a paucity of detailed field performance data, particularly for fish. This has largely been due to the expense and technical challenges associated with underwater videography and volume calibration for trajectory tracking. The increased availability of relatively inexpensive video cameras with underwater housings, in combination with tools for calibration and object tracking, has relaxed these constraints. Collection of field performance data should therefore be prioritized for a broad range of fish species and environmental contexts.

The field performance data collected in the present study indicate the potential value of this approach. The mismatch between field performance and the experimental conditions under which the available energetic data were collected suggests a need to re‐evaluate approaches to quantifying metabolic cost. Estimates of sustained energy expenditure in fish are primarily derived from indirect calorimetry where the rate of oxygen consumption within a sealed volume of water is used as a proxy for metabolic rate. The restricted volumes of recirculating flumes and small, static tanks are required to maximize the temporal resolution of changes in oxygen concentration (Ellerby & Herskin, 2013; Steffensen, 1989). An unconstrained measure of volitional swimming costs would require an alternative cost measurement approach that relaxed the volume constraints associated with closed‐system respirometry.

Alternative physiological approaches to the problem are unfortunately limited. Open‐system respirometry of the type used in air‐breathers is precluded by mechanical complexity of gill ventilation (Strother, 2013). The doubly‐labeled water technique has been employed to measure FMR in air‐breathing vertebrates, but high water turnover rates prevent its use in fish (Brodeur, Dixon, & McKinley, 2001). Mechanical indicators of ventilator cycle frequency may however serve as indicators of respiratory exchange and metabolic rate (Frisk, Skov, & Steffensen, 2012; Millidine, Metcalfe, & Armstrong, 2008; Payne et al., 2011). Cardiac output should also be rapidly responsive to metabolic demand, potentially indicating changes in metabolic effort with respect to volitional swimming maneuvers with a high temporal resolution. Theoretically therefore, if metabolic cost was calibrated to cardiac output using conventional closed‐system respirometry, this relationship could then be applied during volitional swimming (Thorarensen, Gallaugher, & Farrell, 1996; Webber, Boutilier, & Kerr, 1998). Cardiac output is measured using an implanted Doppler flow cuff, so would require tethering or telemetry to transmit the data (Priede & Young, 1977). Despite this, it could allow cost to be estimated during volitional swimming with limited volume constraints that allowed a closer match to field conditions than closed‐system respirometry. Limitations exist however as cardiac output and oxygen consumption are decoupled by changes in blood oxygen extraction, and cost‐cardiac output relationships are potentially shifted by stress or changes in temperature (Brodeur et al., 2001). This requirement for extensive validation within a given species combined with the technical challenges has limited the use of this approach. It may be more practical therefore to modify existing approaches to closed‐system respirometry.

Experimental designs that limit water volume but allow for nonlinear trajectories or velocity variation would allow relationships between the degree of mechanical variation and energetic cost to be quantified. One potential approach would be to re‐adopt the use of annular tank designs that were the basis for early swimming flume designs (Bainbridge, 1958). These were largely abandoned, precisely because of their nonlinearity and the associated elevation of swimming cost (Weihs, 1981). Curved channels would however allow the effects of trajectory curvature on energy expenditure to be directly quantified. A further advantage of this approach is that it offers the possibility of varying fish velocity over relatively short timescales. The inertia of the recirculating water limits how rapidly changes in flow velocity can be achieved in a flume (Ellerby & Herskin, 2013). Some annular tank studies have used a rotating, opaque cover under which the fish seeks shelter as a stimulus for swimming and to control speed (Muir & Niimi, 1972). Such an approach would not be inertia limited and would allow the cost effects of velocity variation on timescales that reflect those in the field to be quantified.

A further issue potentially impacting experimental design is that field swimming behavior differed from previous experimental data not only in terms of measures of mechanical variability, but also regarding the modes of propulsion employed at comparable speeds. Many fish transition through a series of distinct gaits as their speed changes (Webb & Fairchild, 2001). Bluegills have three gaits during flume swimming: MPF swimming powered by the pectoral fins, steady BCF swimming powered by the slow myotomal muscle, and fast, unsteady BCF swimming powered by intermittent bursts of fast myotomal muscle activity (Jones, Lucey, & Ellerby, 2007; Kendall et al., 2007). Defined gait transitions are less apparent in the field (Figure 5). MPF swimming is restricted to low speeds as in the flume (Figure 5). However, it is primarily used for station holding and low‐speed maneuvering rather than for sustained locomotion. The two BCF gaits were employed across the entire speed range, including lower speeds at which the available pectoral girdle muscle power would be adequate to provide thrust (Figure 5).

This mismatch, particularly in terms of the prevalence of low‐speed intermittent propulsion in the field, may be associated with flume spatial constraints. The propulsive phase of intermittent propulsion requires space for forward movement followed by relative backward movement with the flow during the glide phase. If flume working sections are insufficiently long to accommodate this cycle, steady swimming behavior may be imposed (Tudorache, Viaenen, Blust, & DeBoeck, 2007). The effects of intermittent propulsion on swimming cost and the implications for estimating FMR remain unclear. Intermittency may however reduce energy expenditure relative to steady‐state swimming at the same average speed. This is due to the higher drag experienced during propulsion relative to nonpropulsive gliding (Videler & Weihs, 1982; Weihs, 1974). By restricting propulsion to short bursts, the overall cost of producing thrust to overcome drag is potentially reduced. The physiological and mechanical properties of muscle are also relatively constrained, particularly in terms of effective and economical shortening velocities (Marsh, 1999). Intermittency could allow a muscle to meet changing mechanical demands while still optimizing its mechanical performance (Pennycuick, 2001; Rayner, 1985) or reducing contractile costs (Usherwood, 2016). A further priority should therefore be to establish the factors underlying disparities in propulsive mode between volitional and flume swimming at sustained speeds and to determine the implications for estimating the metabolic cost of swimming.

## 5 CONCLUSIONS

Video analyses provide detailed information concerning fish swimming mechanics in the field. This information is a prerequisite for applying laboratory performance data in field activity cost estimates or assessing the fitness implications of particular performance traits. For bluegills sunfish, field video indicated that activity cost estimates based on steady‐state swimming and models based on energetic data obtained during volitional swimming were inapplicable. Measures of how far field behavior deviate from steady‐state conditions may however allow field activity costs to be estimated from steady‐state energetic data. Disparities in field and laboratory swimming behavior suggest that collection of field performance data should be prioritized, and that techniques for quantifying swimming performance in the laboratory need re‐evaluation and potential modification to ensure that they accommodate propulsive behaviors that match those observed in the field.

## CONFLICTS OF INTEREST

None declared.

## AUTHOR CONTRIBUTION STATEMENT

Joanna Milton and Selina Shin contributed to the development, calibration, and deployment of the field video camera rig. Selina Shin and Kelsey Cathcart performed the video analyses. David Ellerby was primarily responsible for statistical analyses and drafting the manuscript.
